# Estimating the Effects of Continuous Albuterol Dosage on Clinical Outcomes for Pediatric Critical Asthma Exacerbation: A Retrospective Cohort Study

**DOI:** 10.1002/ppul.71293

**Published:** 2025-09-11

**Authors:** Daniel P. Riggins, Eneida A. Mendonca, Lucas Bulgarelli, Patricia Tachinardi, Wanzhu Tu, Colin Rogerson

**Affiliations:** ^1^ Center for Biomedical Informatics Regenstrief Institute Indianapolis Indiana USA; ^2^ Department of Epidemiology, Fairbanks School of Public Health Indiana University Indianapolis Indiana USA; ^3^ Division of Biomedical Informatics Cincinnati Children's Hospital Medical Center Cincinnati Ohio USA; ^4^ Department of Pediatrics, College of Medicine University of Cincinnati Cincinnati Ohio USA; ^5^ Department of Biostatistics & Health Data Sciences, School of Medicine Indiana University Indianapolis Indiana USA; ^6^ Indiana University Center for Aging Research Regenstrief Institute Indianapolis Indiana USA; ^7^ Department of Pediatrics Indiana University School of Medicine Indianapolis Indiana USA

## Abstract

**Introduction:**

Pediatric asthma exacerbations sometimes require aggressive intervention including continuous albuterol, a cornerstone therapy for reversing airway obstruction. However, pediatric dosing typically follows adult guidelines, with limited evidence for specific dosing ranges. This study aimed to compare the clinical outcomes of a reduced initial dose (10 mg/h) of continuous albuterol with a standard dose (15 mg/h) in children hospitalized for critical asthma exacerbations.

**Methods:**

This retrospective cohort study was conducted at Riley Hospital for Children, analyzing pediatric patients (ages: 2–18) admitted with critical asthma exacerbations between 2014 and 2022. Stabilized inverse probability weighting (SIPW) was used to adjust for confounding factors between groups. The primary outcome was the percentage change in the Pediatric Asthma Severity Score (PASS) at 24 h, with secondary outcomes including the duration of continuous albuterol, PICU length of stay, and hospital length of stay.

**Results:**

Analysis of 1,486 encounters revealed no significant difference in percent PASS changes at 24 h between the 10 mg/h and 15 mg/h groups (Additive Treatment Effect of 1.69, 95% CI: −0.93–4.31, *p* = 0.207). PICU and hospital lengths of stay were also similar. However, the 10 mg/h group showed a significantly shorter duration of continuous albuterol therapy compared to the 15 mg/h group (Multiplicative Treatment Effect of 1.29, 95% CI: 1.14–1.45, *p* < 0.001).

**Conclusions:**

Findings suggest that starting continuous albuterol at 10 mg/h provides clinical outcomes comparable to 15 mg/h in managing pediatric critical asthma exacerbations. A lower starting dose may optimize resource use and reduce treatment‐related adverse effects.

## 1 Introduction

Asthma remains one of the most prevalent chronic diseases among children worldwide, leading to significant morbidity and occasional mortality. The management of asthma exacerbations often requires interventions that will promptly reverse airway obstruction and prevent further deterioration [1]. Among these interventions, continuous albuterol inhalation stands as one of only two cornerstone therapies, the other being systemic corticosteroids, for which there is a robust evidence base to support its use [2]. Further, albuterol is the only one of these two therapies with rapid‐onset effect. Albuterol is a short‐acting beta‐agonist that works by rapidly relaxing bronchial muscle tissue to improve airway flow and relieve respiratory symptoms [3].

Historically, the dosages of albuterol used in acute settings have been based more on empirical evidence than stringent, dose‐ranging studies. Additionally, albuterol dosing for children is typically derived from adult‐based guidelines using absolute numbers rather than relative to weight. This has led to wide variation in practice, with some clinicians administering higher doses in hopes of quicker resolution of symptoms, despite potential risks such as hypokalemia, tachycardia, cardiac arrhythmias, hypotension, and increased hospital stays due to side effects [4].

Preliminary research in the pediatric population suggests that high‐dose albuterol (75–100 mg/h) does not lead to clinically significant adverse effects [5]. However, a study comparing a broad‐range of weight‐based quintiles did not find evidence for a dose‐effect that would support giving a specific amount for optimal clinical outcomes [6]. Use of continuous albuterol (vs. intermittent) is also associated with increased economic cost to health systems [7, 8]. That, coupled with recently observed critical shortages of continuous albuterol formulations, highlights the need for judicious stewardship of its use [9].

To address these concerns, we conducted an examination of the outcomes associated with administration of a reduced starting dosage of continuous albuterol in children experiencing critical asthma exacerbations. We took advantage of a natural experiment where a change in departmental policy for the pediatric intensive care unit at our institution prompted a change from high variability in the starting rate of continuous albuterol (between 10 and 15 mg/h) to a standardized approach of starting at 10 mg/h. We hypothesized that this lower dose would not lead to worse clinical outcomes.

Our objective was to compare the clinical outcomes for children hospitalized for severe acute asthma treated with starting dose of 10 mg/h of continuous albuterol versus 15 mg/h. Our primary outcome was relative change in clinical asthma symptoms as measured by the Pediatric Asthma Severity Score (PASS). Secondary outcomes included length of time on continuous albuterol, PICU length of stay, and hospital length of stay.

By investigating these outcomes, this study aimed to bolster evidence that lower starting doses of continuous albuterol led to comparable treatment outcomes while opening opportunities for reduced adverse medication side effects and improved hospital resource use.

## 2 Methods

### 2.1 Study Design and Population

We designed this study to estimate the effect of initial continuous albuterol dosage on clinical outcomes for children hospitalized with critical asthma.

We used a cohort of children admitted to a single, quaternary care, academic pediatric center, Riley Hospital for Children, in Indianapolis, IN from January 2014 through December 2022. This study was reviewed by the Indiana University IRB (#17481) and was determined to be Human Subjects Research Exempt. No informed consent was obtained or required since the study met criteria for Human Subjects Research Exemption.

The study selected participants who met specific inclusion criteria. First, subjects were required to be between the ages of 2 and 18 at the time of their admission. Although there are likely children under age 2 that suffer from asthma, we chose to use the traditional clinical definition of a minimum age of 2 years for a formal asthma diagnosis to maintain homogeneity in our population. Second, subjects needed to have a diagnosis of asthma, which was identified using a deterministic computational phenotype including an International Classification of Disease 9th or 10th revision diagnostic code. Third, subjects were required to have received continuous albuterol within the initial 24 h of their admission. Last, subjects were required to have received an oral or intravenous dose of systemic corticosteroids, such as methylprednisolone or prednisone within the first 24 h of their admission. Exclusion criteria included having had a current tracheostomy at the time of admission or having been admitted to the hospital for less than 12 h. Each observation in the data set corresponded to a single encounter. The same child could be observed in multiple encounters. Findings from this study were reported according to the STROBE and TRIPOD guidelines [10, 11].

### 2.2 Data

The data set was derived from the IU Health Enterprise Data Warehouse stewarded for research by Regenstrief Data Services. IU Health is the health system to which Riley Hospital for Children belongs. The initial data was extracted from the data warehouse using Structured Query Language scripts by the IU Health research team. These scripts queried for encounters with a diagnostic code for asthma, or a receipt of albuterol. The cohort was then refined by the study investigators using R [12]. Information was securely stored on a data server located within the Regenstrief Institute. During the extraction process, all sensitive health information was stripped away except for admission dates, discharge dates, birth dates, and home addresses.

### 2.3 Treatment, Outcomes, and Other Variables

The general practice at our institution is for patients to receive three doses of combined albuterol 2.5 mg and ipratropium 0.5 mg within an hour plus systemic steroids at initial presentation. If a patient continues to experience severe symptoms; they are started on continuous albuterol, which is administered via an Aerogen vibrating mesh nebulizer. We determined treatment assignment from the first recorded dose of continuous albuterol for each encounter (10 vs. 15 mg/h). Even if a child was increased to a higher dose later in their hospital course, the initial dose was still used for treatment effect estimation. Our institution's practice was not standardized for the early portion of the study, and most patients were generally started at either 10 or 15 mg/h depending on provider preference. However, in early 2022 due to the national albuterol shortage, we instituted a protocol to start continuous albuterol at 10 mg/h in our intensive care unit. This change in practice is what prompted the study. Before 2022, 70% of encounters received 15 mg/h as the initial dose of continuous albuterol, and 30% received 10 mg/h. After the protocol change, close to 100% of encounters received 10 mg/h as the initial dose. While most patients who receive continuous albuterol in our institution do so in the PICU, there is a small subset who can receive this treatment on the floor, which is consistent with other institution's practice [13].

The primary outcome was the percent change in Pediatric Asthma Severity Score (PASS) measured from index hour 0 to index hour 24. PASS is calculated during periodic assessments by respiratory therapists using a combination of objective and subjective metrics [12]. Secondary outcomes included length of continuous albuterol administration, length of PICU admission, and length of hospital stay. Our hospital uses a protocol based on the PASS for how to wean continuous albuterol. A score of 12–15 triggers an escalation of therapy, a score of 8–11 prompts continuation of current therapy, and a score of 7 or less triggers weaning of therapy. Thus, the minimal clinically important difference would be a difference between the groups of 1 point decrease or greater.

Other data used for descriptive statistics or modeling encompassed a range of patient variables, including demographic information (age, sex, race, and ethnicity), diagnostic data (concurrent pneumonia diagnosis, bronchopulmonary dysplasia, and other chronic obstructive lung disease), vital signs recorded at start of encounter (weight, heart rate, respiratory rate, temperature, oxygen saturation, systolic blood pressure, and diastolic blood pressure), respiratory data (device type and fraction of inspired oxygen), and medication data (ipratropium, magnesium, and epinephrine).

### 2.4 Inverse Probability Weighting

To account for potential confounding and selection bias in the estimation of treatment effects, we used stabilized inverse probability weighting (SIPW) based on propensity score [14]. The propensity score was defined as the probability of receiving 15 mg/h of continuous albuterol (vs. 10 mg/h) conditional on a set of baseline covariates. The code for SIPW was informed by course materials written in Heiss [15].

### 2.5 Propensity Score Model

A parsimonious set of baseline covariates was selected for use in estimating the propensity score using AIC‐based stepwise selection (both forward and backward) [16]. The covariates selected were year of admission, patient's age at admission, whether the patient identified their race as Black/African American, patient's initial heart rate, and patient's initial respiratory rate.

### 2.6 Treatment Effect Models

SIPW was used to adjust our treatment effect models. Treatment effect on the primary outcome (percent PASS change at 24 h) was estimated using simple linear regression. Treatment effects on the secondary length‐of‐time outcomes were estimated using negative binomial generalized linear models. Adjusted effect estimates and 95% confidence intervals were reported. All statistical tests were two‐sided, and a *p*‐value less than 0.05 was considered statistically significant.

### 2.7 Post‐Hoc Sub Analysis

We conducted a post‐hoc sub analysis stratifying patients based on age. There was a concern that younger patients were more likely to receive a higher dose per kilogram of continuous albuterol using our hospital protocols, and if age were unbalanced between the groups this could bias the study outcomes. The cohort divided into two with one group containing encounters ages 2–6 years, and another containing encounters ages 7–18 years. Each group was evaluated for the total dose of albuterol received during hospitalization in mg/kg/h, and each stated study outcome.

### 2.8 Software and Data Availability

Data analysis was performed using the R programming language in RStudio [12, 17]. R packages used in the analysis included “gt,” “gtsummary,” “MASS,” “parameters,” “performance,” “ranger,” “targets,” “tidymodels,” and “tidyverse” [16, 18, 19, 20, 21, 22, 23, 24, 25].

Due to the presence of personally identifiable information, the data supporting this study are not openly available. However, the data are available from the corresponding author upon reasonable request and subject to appropriate ethical approvals and data sharing agreements. Interested researchers are invited to submit inquiries along with a detailed proposal for use.

## 3 Results

### 3.1 Baseline Characteristics

We analyzed the baseline characteristics of both the 10 and 15 mg/h initial continuous albuterol treatment groups. The sample included 1486 encounters, of which 575 received a starting dose of 10 mg/h and 911 received the 15 mg/h. There were 93 encounters where treatment initiated at 10 mg/h but subsequently increased to 15 mg/h (23.3% of the 10 mg/h treatment group, 9% of all encounters). Of the 1486 included encounters, 324 (21.8%) of encounters were repeated encounters for the same individual.

Table 1 summarizes demographic and clinical variables, such as age, sex, baseline health status, and initial vital signs while also comparing between the 10 and 15 mg/h treatment groups. For all encounters, characteristics included median age of 7 years (IQR: 4–10), 57% Black race, 59% male, 84% involving a PICU admission, and median hospital length of stay of 2.8 days (IQR: 2.1–3.9). For the primary outcome, 435 (29.3%) of the encounters did not have a PASS measured at 24‐h.

**Table 1:** Baseline demographic and clinical characteristics.

		Initial continuous albuterol dose		
Characteristic	Overall, *N* = 1486	10 mg/h, *N* = 575	15 mg/h, *N* = 911	*p* value^1^
**Demographics**				
Age (yrs), Median (IQR)	7 (4–10)	4 (3–7)	8 (6–12)	< 0.001
Sex, *n* (%)				0.37
Female	608 (41)	227 (39)	381 (42)	
Male	878 (59)	348 (61)	530 (58)	
Race, *n* (%)				< 0.001
Asian	18 (1.2)	11 (1.9)	7 (0.8)	
Black or African American	831 (57)	290 (51)	541 (61)	
White	603 (41)	263 (46)	340 (38)	
Other	3 (0.2)	2 (0.4)	1 (0.1)	
Missing	31	9	22	
Ethnicity, *n* (%)				0.086
Hispanic or latino	191 (13)	85 (15)	106 (12)	
Not Hispanic or latino	1,276 (87)	485 (85)	791 (88)	
Missing	19	5	14	
**Clinical factors**				
Bronchopulmonary dysplasia, *n* (%)	36 (2.4)	22 (3.8)	14 (1.5)	0.005
Comorbid pneumonia, *n* (%)	310 (21)	137 (24)	173 (19)	0.025
Other Chronic Obstructive Lung Disease, *n* (%)	4 (0.3)	2 (0.3)	2 (0.3)	0.64
**Initial vital signs**				
Temperature, Median (IQR)	37.0 (36.7–37.3)	37.0 (36.7–37.2)	37.0 (36.7–37.3)	0.34
Heart Rate, Median (IQR)	145 (131–158)	148 (134–162)	144 (129–156)	< 0.001
Respiratory Rate, Median (IQR)	32 (26–40)	36 (28–42)	31 (26–39)	< 0.001
Oxygen Saturation, Median (IQR)	96 (94–98)	96 (94–98)	96 (94–98)	0.074
Pediatric Asthma Severity Score, Median (IQR)	9 (7–10)	9 (7–10)	9 (7–10)	0.66
Missing	435	130	305	
Systolic Blood Pressure, Median (IQR)	111 (103–121)	110 (102–118)	113 (103–122)	< 0.001
Diastolic Blood Pressure, Median (IQR)	59 (50–70)	62 (53–71)	58 (49–69)	< 0.001

^1^ Wilcoxon rank sum test; Pearson's Chi‐squared test; Fisher's exact test.

Before applying stabilized inverse probability weighting (SIPW), there were statistically significant differences between the treatment groups. The median age of encounters in the 10 mg/h group was 4 years (IQR: 3–7) compared to 8 (IQR: 6–12) in the 15 mg/h group (*p* < 0.001). The percentage of encounters with the patient identifying as Black in the 10 mg/h group was 51% compared to 61% in the 15 mg/h group (*p* < 0.001). The median initial respiratory rate in the 10 mg/h group was 36 (IQR: 28–42) compared to 31 (IQR: 26–39) in the 15 mg/h group (*p* < 0.001). However, there was not a statistically significant difference in initial PASS between the groups–both having a median of 9 (*p* = 0.66).

### 3.2 Unadjusted Treatments and Outcomes

Table 2 presents treatments and outcomes for all encounters and comparisons between the 10 and 15 mg/h initial treatment groups. For the primary outcome, there was an overall combined median change in PASS at 24 h of −17% (IQR: −31–0), indicating modest clinical improvement for most patients.

**Table 2:** Unadjusted treatments and outcomes.

		Initial continuous albuterol dose		
Characteristic	Overall, *N* = 1486	10 mg/h, *N* = 575	15 mg/h, *N* = 911	*p* value^1^
**Treatments**				
Initial oxygen support, *n* (%)				0.18
BiPAP	18 (1.2)	4 (0.7)	14 (1.5)	
HFNC	140 (9.4)	63 (11)	77 (8.5)	
IMV	33 (2.2)	11 (1.9)	22 (2.4)	
RA/NC	1,295 (87)	497 (86)	798 (88)	
Duration of respiratory support, median (IQR)	24 (10–40)	18 (10–36)	24 (10–46)	0.03
Initial fraction of inspired oxygen, median (IQR)	0.35 (0.21–0.50)	0.36 (0.21–0.60)	0.30 (0.21–0.50)	0.002
Missing	48	20	28	
Weight‐based albuterol dose (mg/kg/h)	0.49 (0.33–0.67)	0.54 (0.42–0.65)	0.47 (0.30–0.70)	0.01
Total continuous albuterol dose (mg)	215 (94–404)	134 (59–272)	284 (141–469)	< 0.001
Ipratropium ever given, *n* (%)	1202 (81)	460 (80)	742 (81)	0.49
Magnesium ever given, *n* (%)	1239 (83)	465 (81)	774 (85)	0.039
Magnesium bolus doses, median (IQR)	1.0 (1.0–2.0)	1.0 (1.0–1.0)	1.0 (1.0–2.0)	< 0.001
Epinephrine ever given, *n* (%)	49 (3.3)	5 (0.9)	44 (4.8)	< 0.001
**Outcomes**				
PASS (% change at 24 h), median (IQR)	−17 (−31–0)	−19 (−33–0)	−17 (−30–0)	0.11
Missing	438	131	307	
Length of continuous albuterol therapy (hrs), median (IQR)	16 (7–29)	13 (6–24)	19 (9–31)	< 0.001
Length of PICU stay (days), median (IQR)	1.5 (1.0–2.2)	1.4 (1.0–2.2)	1.5 (1.0–2.2)	0.30
Missing	245	88	157	
Intubation, *N* (%)	67 (4.5)	23 (4.7)	44 (4.5)	0.98
Length of hospital admission (days), median (IQR)	2.8 (2.1–3.9)	2.7 (2.0–3.9)	2.8 (2.2–4.0)	0.046

^1^ Pearson's Chi‐squared test; Wilcoxon rank sum test.

**Figure 1:**
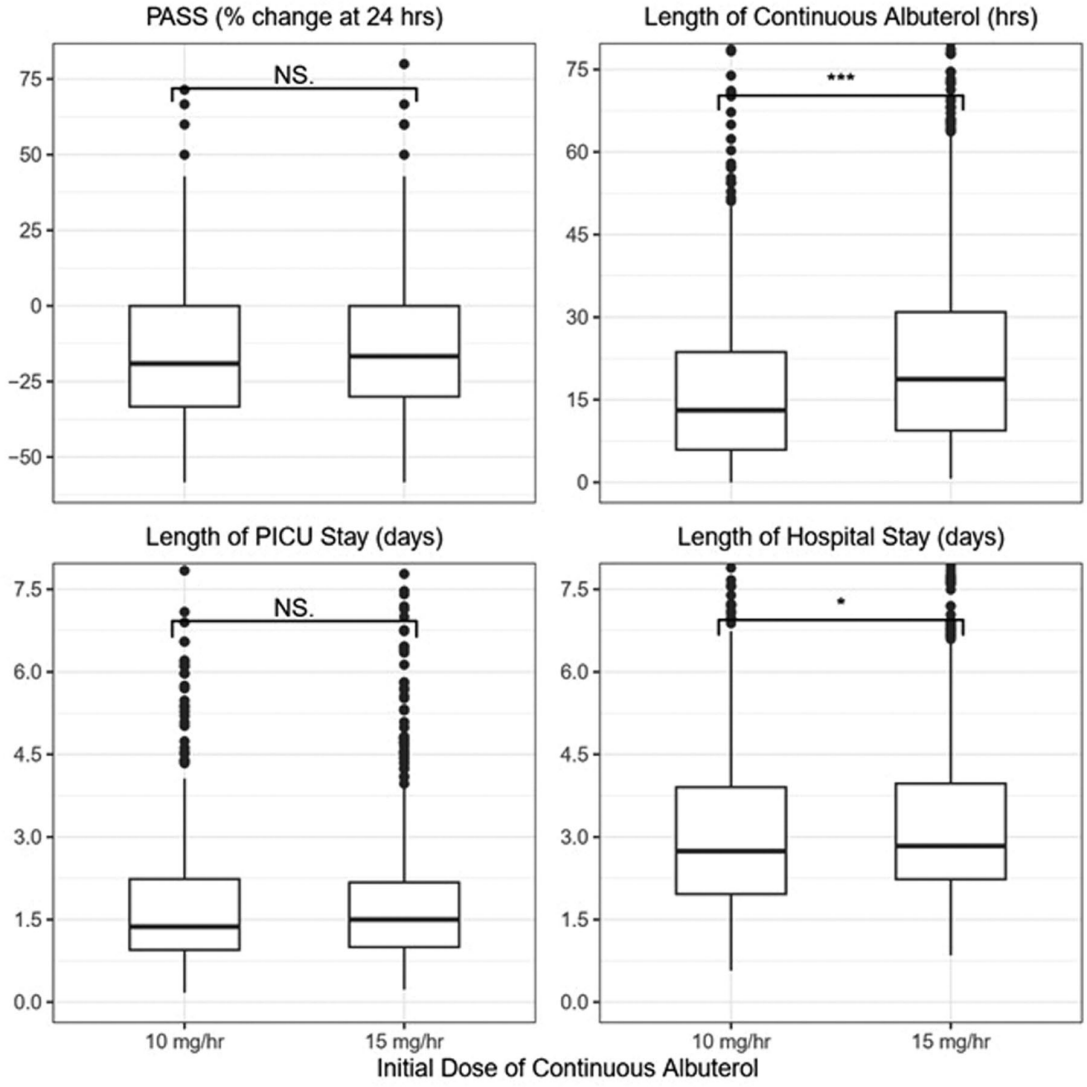
Boxplots comparing percent change in PASS at 24 h, length of continuous albuterol in hours, length of PICU stay in days, and length of hospital stay in days between the 10 mg/h continuous albuterol group on the left, and the 15 mg/h group on the right.

Before applying SIPW, there was no significant difference observed between treatment groups on the primary outcome, but there were significant differences on two of the secondary outcomes (Figure 1). The median length of continuous albuterol therapy in the 10 mg/h group was 13 h (IQR: 6–24) compared to 19 h (IQR: 9–31) in the 15 mg/h group (*p* < 0.001). The median length of hospital stay in the 10 mg/h group was 2.7 days (IQR: 2.0–3.9) compared to 2.8 (IQR: 2.2–4.0) in the 15 mg/h group (*p* = 0.046).

### 3.3 Propensity Score Estimation

Supplemental Report 1 describes and shows code for a comparison of two different models used to assign propensity scores. Upon evaluation, a random forest model using a parsimonious set of covariates (year, age, Black race, initial heart rate, and initial respiratory rate) performed best with accuracy of 0.73 and AUC of 0.76 at predicting the observed treatment group.

### 3.4 Assessment of SIPW Covariate Balance

We assessed the balance of covariates used for propensity score estimation before and after applying stabilized inverse probability weighting. Before weighting, all covariates were significantly unbalanced (standardized mean difference > 0.1). After applying SIPW, all covariates except age (SMD = 0.36) achieved acceptable balance (SMD < 0.1).

### 3.5 Main Analysis: Treatment Effect Estimation

Using SIPW, we applied regression models to estimate the effect of starting continuous albuterol dosage on our chosen outcome measures (Table 3). Consistent with unadjusted comparisons, the weighted analysis did not show a significant treatment effect on the primary outcome—percent change in PASS at 24 h (95% CI: −0.93–4.31, *p* = 0.207). Also consistent with the unadjusted comparisons, the weighted analysis did show a significant treatment effect on length of continuous albuterol administration, estimating that receiving an initial dose of 15 mg/h (vs. 10 mg/h) multiplied the length of continuous albuterol by 1.29 h (95% CI: 1.14–1.45, *p* < 0.001). Unlike the unweighted comparison, the weighted analysis did not show a significant treatment effect on length of hospital stay (95% CI: 0.9–1.04, *p* = 0.373).

**Table 3:** Treatment effect of 15 mg/h initial dose (vs. 10 mg/h).

Treatment effect of 15 mg/hr initial dose (vs. 10 mg/hr)					
Outcome	Effect	Confidence interval	*p* value	Effect type	Model type
PASS (% change at 24 h)	1.69	−0.93–4.31	0.207	Additive	Simple linear
Length of continuous albuterol	1.29	1.14–1.45	< 0.001	Multiplicative	Negative binomial
Length of PICU stay	1.00	0.91–1.11	0.987	Multiplicative	Negative binomial
Length of hospital stay	0.97	0.9–1.04	0.373	Multiplicative	Negative binomial

### 3.6 Sensitivity Analysis

We conducted sensitivity analyses to examine the robustness of our findings under different propensity score models and weighting schemes. The results remained consistent across all scenarios, supporting the robustness of our findings.

### 3.7 Age‐Stratified Sub Analysis

The sub analysis based on age had a total of 740 encounters ages 7–18, and 715 encounters ages 2–6 (Tables 4a and 4b). There was a difference in distribution based on treatment group, with 80% of the older group receiving 15 mg/h of continuous albuterol, while only 41% of the younger group received this dose. In both age groups, the treatment group (10 mg/h) received less total continuous albuterol in mg/kg/h. In the older age group, while there were no differences in PASS score change, PICU length of stay, or hospital length of stay, there was a significant difference in length of time on continuous albuterol, with the treatment group receiving less. In the younger age group, there were no significant differences in any of the study outcomes.

**Table 4a:** Weight‐based Albuterol dosing and Treatment Effect of 15 mg/hr Initial Dose (vs. 10 mg/hr) in children age 7–18 years.

Characteristic	Overall, *N* = 740	10 mg/h, *N* = 147	15 mg/h, *N* = 593	*p* value
Weight‐based albuterol dose (mg/kg/hr)	0.34 (0.18–0.64)	0.20 (0.09–0.37)	0.31 (0.17–0.54)	<0.001

Treatment effect			
Outcome	Effect	Confidence interval	*p* value
PASS (% change at 24 h)	−1.54	−2.27–5.34	0.428
Length of continuous albuterol	1.53	1.29–1.81	< 0.001
Length of PICU stay	1.09	0.94–1.26	0.25
Length of hospital Stay	0.99	0.89–1.10	0.85

**Table 4b:** Weight‐based Albuterol dosing and Treatment Effect of 15 mg/h Initial Dose (vs. 10 mg/h) in children age 2–6 years.

Characteristic	Overall, *N* = 715	10 mg/h, *N* = 419	15 mg/h, *N* = 296	*p* value
Weight‐based albuterol dose (mg/kg/hr)	0.42 (0.21–0.78)	0.35 (0.17–0.69)	0.50 (0.30–0.87)	< 0.001

Treatment effect			
Outcome	Effect	Confidence interval	*p* value
PASS (% change at 24 h)	1.32	−2.36–5.01	0.48
Length of continuous albuterol	1.02	0.87–1.21	0.80
Length of PICU stay	0.90	0.79–1.04	0.15
Length of hospital stay	0.92	0.83–1.02	0.12

## 4 Discussion

In this retrospective cohort study evaluating outcomes of pediatric patients with severe acute asthma, we compared two separate starting doses of continuous albuterol. We found that there was no significant difference in clinical symptom course between the two groups. We also found that there was no difference in hospital or PICU LOS. There was a significant difference in the length of time spent on continuous albuterol between the two groups.

### 4.1 Interpretation of Findings

Reducing the initial dosage of continuous albuterol from 15 to 10 mg/h did not result in discernible changes in Pediatric Asthma Severity Score (PASS) at 24 h, suggesting that lower starting doses of continuous albuterol may be equally effective in managing critical asthma exacerbations in children.

Furthermore, the reduction in the median length of continuous albuterol therapy in the lower dosage group indicates potential resource‐saving opportunities associated with lower dosing regimens. We also saw a lower total dose of continuous albuterol in the treatment group, as well as significantly lower total time on respiratory support.

The age‐stratified sub analysis revealed that the treatment group did receive a lower weight‐based dose of continuous albuterol, despite the opposite difference seen in the full cohort. This indicates that the lower initial dose protocol leads to less overall exposure to albuterol in both flat rate dosing and weight‐based dosing. With this lower dosing, the treatment group in both age groups had no significant difference in clinical outcomes that would indicate they were receiving a suboptimal dose of continuous albuterol.

### 4.2 Comparison With Existing Literature

To our knowledge, no other study has used the same primary outcome or the same comparison of dosage amounts that this study has. Leveraging access to a database constructed using cutting‐edge informatics tools, we were able to look at granular clinical symptoms like the PASS, heart rate, and respiratory rate, which are extremely valuable to the clinical context of asthma research.

The most comparable study was Lin et al. which was also a retrospective cohort study comparing 10 to 25 mg/h of continuous albuterol and found results overall consistent with our own [26]. However, perhaps because of the wider comparison margin, they were able to detect more differences in outcomes showing that the lower dose group did not require more adjunctive therapies but did have reduced need for fluid resuscitation and shorter PICU/hospital lengths of stay. Our study adds value to this by adding an additional level of dosage for comparison and using a more direct measure of clinical status as our primary outcome (change in PASS at 24 h).

Parlar‐Chun and Arnold was also a retrospective cohort study looking at a similar set of outcomes to our own such as asthma severity score, length of continuous albuterol administration, and length of stay, but it is difficult to directly compare findings since they looked at weight‐based dosage quintiles [6]. That said, the total range of raw doses they evaluated was 5–20 mg/h and they were unable to detect advantages for any dosage quintile group across their selected outcomes. We calculated the weight‐based dosing of albuterol and found that both groups had median dosing close to the standard recommended 0.5 mg/kg/h [6]. It could be argued that rather than standardizing a specific dosing amount, such as 10 mg/h, a better approach may be using weight‐based dosing of 0.5 mg/h, but further study would need to test this hypothesis.

Finally, Stein and Levitt performed a small‐scale RCT (127 subjects) comparing 7.5 to 15 mg/h and were unable to find differences in clinical outcomes like vital signs, dyspnea scale, peak flow, or length of stay in the ED [27]. It is hard to definitively say whether they were unable to detect a difference because of small sample sizes and/or because of the different clinical context (ED vs. inpatient), but their results do not directly contradict any of our Findings.

### 4.3 Significance of Findings

First and foremost, our study provides valuable insights into the potential reduction of medication‐related side effects associated with lower dosages of continuous albuterol in children hospitalized for severe acute asthma exacerbations. While we did not specifically investigate side effects in this study, it is reasonable to infer that a lower dosage and shorter duration of continuous albuterol therapy may lead to fewer adverse effects. As healthcare providers, our primary obligation is to prioritize patient safety and minimize harm. Therefore, the identification of a potentially safer and equally effective dosage regimen aligns with our ethical commitment to “do no harm.”

Second, while the observed differences in clinical outcomes may appear modest within our cohort, it is essential to recognize the broader impact of optimizing treatment strategies for pediatric asthma, particularly in the context of the pediatric intensive care unit (PICU). Asthma exacerbations are a prevalent and recurring condition seen not only in our institution but in PICUs across the country and around the world. By identifying potential avenues for improving asthma management, even if the benefits per patient are incremental, we make a substantial collective impact on the well‐being of millions of children globally.

This study represents a step towards precision medicine for children hospitalized for critical asthma. We found that, in general, our cohort of patients had similar clinical outcomes with a lower dose of continuous albuterol. It is likely though that certain subgroups of patients have differential response to treatment, including some patients that have severe disease and respond better to a higher dose (15 mg/h), and others that may be either less severe or more prone to side effects and have better outcomes at a lower dose (10 mg/h). Additional future studies with larger, more robust datasets from multiple institutions are needed to further refine the practice of precision medicine in this population.

### 4.4 Strengths and Limitations

One of the strengths of our study is the use of stabilized inverse probability weighting (SIPW) to adjust for potential confounding factors and selection bias, enhancing the robustness of our results. Another strength of our study is the use of change in PASS as our primary outcome, which gives a composite representation of the patient's clinical status in the domains most relevant to critical assessment of asthma.

However, our study also has limitations. Reliance on data from a single academic pediatric center, may limit the generalizability of our findings. While use of SIPW bolstered our causative claims for treatment effects, this is still a retrospective observational study and may be subject to residual bias due to unmeasured confounders. Thus, causality should be interpreted with caution. Less than a quarter of patients assigned to the 10 mg/h treatment group were subsequently increased to 15 mg/h based on clinical status. However, we would expect patients like this to bias the data toward poorer outcomes for the 10 mg/h treatment group, which we did not observe. A significant portion of our cohort were missing the primary outcome of PASS at 24‐h, which may add bias to our conclusions. The difference in age between the groups led to a whole cohort difference in weight‐based albuterol dosing received which may bias the study outcomes. However, the post‐hoc sub analysis revealed that when stratified by age, the treatment group did receive a lower weight‐based dose of continuous albuterol as well as flat rate dosing. While additional therapies such as ipratropium, magnesium sulfate, and epinephrine were included in the analysis, others were not, such as cumulative pre‐hospitalization albuterol or systemic steroids, which may introduce bias. Finally, although relatively limited, there were still missing observations for some outcomes, which could also add bias to the results.

### 4.5 Future Directions

Future research could follow several different threads of inquiry. First, studies could replicate our findings in diverse clinical settings and populations to validate the effectiveness of 10 mg/h of initial continuous albuterol in pediatric asthma management. Second, studies could investigate even lower dosages, a broader panel of dosages, and/or weight‐based dosing regimens to see if comparable outcomes persist. Also, studies could further investigate the long‐term outcomes and possible adverse effects associated with lower doses of continuous albuterol. Finally, prospective, randomized trials would be most effective in determining the true treatment effects of separate dosing regimens.

## 5 Conclusion

In conclusion, our study contributes to the understanding of inpatient pediatric asthma management by providing evidence that lower starting doses of continuous albuterol may be equally effective as higher doses in managing severe acute asthma exacerbations in children. By addressing concerns regarding variability in dosing practices and potential adverse effects, we have advanced knowledge in the field and paved the way for safer care as well as better resource management.

## Author Contributions

**Daniel P. Riggins:** writing – original draft, methodology, visualization, writing – review and editing, formal analysis, investigation. **Eneida A. Mendonca:** writing – review and editing, supervision. **Lucas Bulgarelli:** writing – review and editing, methodology. **Patricia Tachinardi:** writing – review and editing, methodology. **Wanzhu Tu:** methodology, writing – review and editing. **Colin Rogerson:** conceptualization, investigation, methodology, writing – review and editing, formal analysis, data curation, supervision.

## Conflicts of Interest

The authors declare no conflicts of interest.

## Supporting information

supplementary report 1.

## Data Availability

The data that support the findings of this study are available on request from the corresponding author. The data are not publicly available due to privacy or ethical restrictions.
