# Supplementary material for: Estimating the Effects of Continuous Albuterol Dosage on Clinical Outcomes for Pediatric Critical Asthma Exacerbation: A Retrospective Cohort Study
Source: Pediatr Pulmonol. 2025 Sep 11;60(9):e71293. doi: 10.1002/ppul.71293 (PMC12424099; doi:10.1002/ppul.71293)
Supplement: Supplementary file 1 — supplementary report 1. [file PPUL-60-0-s001.html]

Choosing a Model for Assigning Inverse Probability Weights to Continuous Albuterol Treatment


# Choosing a Model for Assigning Inverse Probability Weights to Continuous Albuterol Treatment

Author

Daniel P. Hall Riggins, MD, MPH

Published

October 30, 2024

In this report, we are interested in modeling inverse probability weights for the treatment variable in our main analysis, that is, whether a patient admitted for asthma exacerbation received a peak continuous albuterol dose of 10 vs. 15 mg/hr.

# Libraries and Settings

Code

```
# Standard Libraries
library(targets)
library(tidyverse)
library(tidymodels)
library(parameters)
library(here)
library(janitor)
library(plotly)

# Custom Function
source(here("R", "tabyl_modified.R"))

# Settings
theme_set(theme_bw())
set.seed(1989)
# i_am("reports/propensity_modeling.qmd")
# tar_config_set(store = here("_targets"))

# Starting Data
tar_load(asthmaster_modified)
```

# Explore the Data

Production of this project’s dataset has been specified in a targets pipeline you can view in my Github repository. Note that the raw data itself is not accessible in the repository due to being protected health information.

In the original dataset, there were a variety of peak continuous albuterol doses listed:

Code

```
tar_read(asthmaster_raw) |> 
    tabyl_modified(Albuterol_Cont_Dose)
```

We have limited our analysis to the subset of patient encounters where the peak dose was either 10 or 15 mg/hr and have converted this variable to a factor.

Code

```
asthmaster_modified |> 
    tabyl_modified(Albuterol_Cont_Dose_Factor)
```

We have selected a subset of possible modeling covariates that could plausibly have a causal influence on the probability of receiving treatment. Here we skim the characteristics of these possible covariates:

Code

```
asthmaster_modified |> 
    select(
        Year,
        AgeAtEncounter, 
        Weight.VPS, 
        Weight,
        Race,
        Race_Black, 
        Ethnicity,
        Ethnicity_Hispanic,
        Sex,
        Patient.Origin,
        Ipratropium,
        PneumoniaDx, 
        ChronicLungdzDx, 
        CCC, 
        BPD,
        HR_Initial, 
        RR_Initial, 
        O2_Initial, 
        FiO2_Initial,
        PASS_Initial,
        Temp_Initial,
        Resp_Initial
    ) |> 
    skimr::skim()
```

Data summary

|  |  |
| --- | --- |
| Name | select(…) |
| Number of rows | 1486 |
| Number of columns | 22 |
| \_\_\_\_\_\_\_\_\_\_\_\_\_\_\_\_\_\_\_\_\_\_\_ |  |
| Column type frequency: |  |
| character | 3 |
| factor | 2 |
| logical | 6 |
| numeric | 11 |
| \_\_\_\_\_\_\_\_\_\_\_\_\_\_\_\_\_\_\_\_\_\_\_\_ |  |
| Group variables | None |

**Variable type: character**

| skim\_variable | n\_missing | complete\_rate | min | max | empty | n\_unique | whitespace |
| --- | --- | --- | --- | --- | --- | --- | --- |
| Sex | 0 | 1.00 | 4 | 6 | 0 | 2 | 0 |
| Patient.Origin | 245 | 0.84 | 4 | 98 | 0 | 12 | 0 |
| Resp\_Initial | 0 | 1.00 | 3 | 5 | 0 | 4 | 0 |

**Variable type: factor**

| skim\_variable | n\_missing | complete\_rate | ordered | n\_unique | top\_counts |
| --- | --- | --- | --- | --- | --- |
| Race | 31 | 0.98 | TRUE | 4 | Bla: 831, Whi: 603, Asi: 18, Oth: 3 |
| Ethnicity | 19 | 0.99 | TRUE | 2 | Not: 1276, His: 191 |

**Variable type: logical**

| skim\_variable | n\_missing | complete\_rate | mean | count |
| --- | --- | --- | --- | --- |
| Race\_Black | 31 | 0.98 | 0.57 | TRU: 831, FAL: 624 |
| Ethnicity\_Hispanic | 19 | 0.99 | 0.13 | FAL: 1276, TRU: 191 |
| Ipratropium | 0 | 1.00 | 0.97 | TRU: 1445, FAL: 41 |
| PneumoniaDx | 0 | 1.00 | 0.21 | FAL: 1176, TRU: 310 |
| ChronicLungdzDx | 0 | 1.00 | 0.00 | FAL: 1482, TRU: 4 |
| BPD | 0 | 1.00 | 0.02 | FAL: 1450, TRU: 36 |

**Variable type: numeric**

| skim\_variable | n\_missing | complete\_rate | mean | sd | p0 | p25 | p50 | p75 | p100 | hist |
| --- | --- | --- | --- | --- | --- | --- | --- | --- | --- | --- |
| Year | 0 | 1.00 | 2017.93 | 2.96 | 2014.00 | 2015.00 | 2018.00 | 2021.00 | 2022.0 | ▇▅▂▃▇ |
| AgeAtEncounter | 0 | 1.00 | 7.40 | 4.05 | 2.00 | 4.00 | 7.00 | 10.00 | 18.0 | ▇▆▃▃▁ |
| Weight.VPS | 245 | 0.84 | 34.20 | 22.42 | 9.10 | 18.20 | 26.00 | 44.00 | 149.4 | ▇▂▁▁▁ |
| Weight | 735 | 0.51 | 35.56 | 23.76 | 5.90 | 18.55 | 27.00 | 45.15 | 156.5 | ▇▃▁▁▁ |
| CCC | 0 | 1.00 | 0.12 | 0.51 | 0.00 | 0.00 | 0.00 | 0.00 | 6.0 | ▇▁▁▁▁ |
| HR\_Initial | 0 | 1.00 | 143.64 | 21.02 | 22.00 | 131.00 | 145.00 | 158.00 | 206.0 | ▁▁▃▇▁ |
| RR\_Initial | 0 | 1.00 | 34.27 | 10.73 | 8.00 | 26.00 | 32.00 | 40.00 | 80.0 | ▂▇▅▁▁ |
| O2\_Initial | 0 | 1.00 | 95.76 | 2.94 | 66.00 | 94.00 | 96.00 | 98.00 | 100.0 | ▁▁▁▂▇ |
| FiO2\_Initial | 48 | 0.97 | 0.43 | 0.26 | 0.21 | 0.21 | 0.35 | 0.50 | 1.0 | ▇▃▁▁▂ |
| PASS\_Initial | 435 | 0.71 | 8.60 | 1.77 | 5.00 | 7.00 | 9.00 | 10.00 | 14.0 | ▂▇▇▂▁ |
| Temp\_Initial | 0 | 1.00 | 36.99 | 0.63 | 26.50 | 36.70 | 37.00 | 37.30 | 39.7 | ▁▁▁▇▆ |

The variables Patient.Origin, Weight/Weight.VPS, and PASS\_Initial all have too many missing values to be useful in this scope. The variables Race/Race\_Black, Ethnicity/Ethnicity\_Hispanic, and FiO2\_Initial all have a small amount of missing values. We could theoretically include them in modeling, but it would mean we’d have to throw away some observations, so let’s cheat a little and see how strong their unadjusted associations are with the treatment variable in logistic regression models.

Code

```
c("Race_Black", "Ethnicity_Hispanic", "FiO2_Initial") |> 
    map(
        ~ parameters(
            glm(
                formula(paste("Albuterol_Cont_Dose_Factor ~", .x)),
                data = asthmaster_modified,
                family = binomial
            ),
            exponentiate = TRUE
        )
    ) |>
    reduce(bind_rows) |>
    filter(Parameter != "(Intercept)")
```

It looks like the unadjusted association with Race\_Black variable is strong, so it might be worth throwing away one missing observation to explore further. The other two variables don’t have a strong enough unadjusted association to make it worth throwing away observations to use them.

Let’s create a more compact dataset just containing our treatment variable and prospective covariates for the model with missing observations dropped:

Code

```
asthmaster_compact <-
    asthmaster_modified |> 
    select(
        FIN,
        Albuterol_Cont_Dose_Factor,
        Year,
        AgeAtEncounter, 
        # Weight.VPS, 
        # Weight,
        # Race,
        Race_Black, 
        # Ethnicity,
        # Ethnicity_Hispanic,
        Sex,
        # Patient.Origin,
        Ipratropium,
        PneumoniaDx, 
        ChronicLungdzDx,
        CCC, 
        BPD,
        HR_Initial, 
        RR_Initial, 
        O2_Initial, 
        # FiO2_Initial,
        # PASS_Initial,
        Temp_Initial,
        Resp_Initial
    ) |> 
    na.omit()

asthmaster_compact |> 
    select(-FIN) |>
    glimpse()
```

```
Rows: 1,455
Columns: 15
$ Albuterol_Cont_Dose_Factor <ord> 15 mg/hr, 15 mg/hr, 15 mg/hr, 15 mg/hr, 15 …
$ Year                       <int> 2017, 2014, 2014, 2014, 2014, 2014, 2014, 2…
$ AgeAtEncounter             <int> 12, 8, 5, 13, 10, 16, 2, 3, 8, 10, 2, 12, 7…
$ Race_Black                 <lgl> FALSE, FALSE, FALSE, TRUE, TRUE, TRUE, TRUE…
$ Sex                        <chr> "Female", "Female", "Female", "Male", "Fema…
$ Ipratropium                <lgl> TRUE, TRUE, TRUE, TRUE, TRUE, TRUE, TRUE, T…
$ PneumoniaDx                <lgl> TRUE, FALSE, FALSE, FALSE, FALSE, TRUE, FAL…
$ ChronicLungdzDx            <lgl> FALSE, FALSE, FALSE, FALSE, FALSE, FALSE, F…
$ CCC                        <int> 0, 0, 0, 0, 0, 0, 0, 0, 0, 0, 1, 0, 0, 0, 0…
$ BPD                        <lgl> FALSE, FALSE, FALSE, FALSE, FALSE, FALSE, F…
$ HR_Initial                 <int> 108, 152, 160, 131, 119, 124, 174, 190, 126…
$ RR_Initial                 <int> 28, 36, 34, 40, 21, 24, 46, 48, 41, 25, 40,…
$ O2_Initial                 <int> 98, 96, 100, 98, 100, 98, 96, 97, 100, 96, …
$ Temp_Initial               <dbl> 36.2, 36.8, 36.5, 37.3, 36.4, 37.6, 37.1, 3…
$ Resp_Initial               <chr> "RA/NC", "RA/NC", "RA/NC", "RA/NC", "RA/NC"…
```

# Model and Evaluate

Now let’s split our dataset into train and test sets then draw some bootstrap samples from the training set:

Code

```
asthmaster_split <- 
    initial_split(
        asthmaster_compact, 
        prop = 0.75, 
        strata = Albuterol_Cont_Dose_Factor
    )
asthmaster_train <- training(asthmaster_split)
asthmaster_test <- testing(asthmaster_split)

asthmaster_boot <- bootstraps(asthmaster_train)
```

We are going to try out two different models based on different predictor sets–these being all predictors vs. a parsimonious subset chosen by clinical judgement and unadjusted significance testing (year, age, Black race, initial heart rate, and initial respiratory rate).

Here we specify the generic random forest model. Note that we will be tuning the mtry and min\_n hyperparameters:

Code

```
rf_spec <-
    rand_forest(
        mode = "classification",
        engine = "ranger",
        trees = 500,
        mtry = tune(),
        min_n = tune()
    )
```

And specify our predictor sets:

Code

```
all_pred_spec <- formula("Albuterol_Cont_Dose_Factor ~ . - FIN")

subset_pred_spec <- formula("Albuterol_Cont_Dose_Factor ~ Year + AgeAtEncounter + Race_Black + HR_Initial + RR_Initial")
```

Fitting random forest models while tuning hyperparameters is a computationally expensive process so I have run this ahead of time and load the results into this report. As a quick aside, let’s visualize different combinations of hyperparameters (mtry and min\_n) to see how they impact performance (mean\_roc\_auc). The interactive 3D plot below shows that a combination of mtry = 2 and min\_n = 36 have created the best performing model on average with mean\_roc\_auc = 0.75 (you may need to rotate the plot a little to see this clearly):

Code

```
# rf_all <-
#     workflow() |>
#     add_model(rf_spec) |>
#     add_formula(all_pred_spec) |>
#     tune_grid(
#         resamples = asthmaster_boot,
#         grid = 25
#     )
# 
# rf_subset <-
#     workflow() |>
#     add_model(rf_spec) |>
#     add_formula(subset_pred_spec) |>
#     tune_grid(
#         resamples = asthmaster_boot,
#         grid = 25
#     )
```

Code

```
rf_all <- tar_read(tunes_ipw_all_rf)
rf_subset <- tar_read(tunes_ipw_subset_rf)

rf_subset |> 
    collect_metrics() |>
    filter(.metric == "roc_auc") |>
    select(mean_roc_auc = mean, min_n, mtry) |> 
    plot_ly(
        x = ~min_n,
        y = ~mtry,
        z = ~mean_roc_auc,
        type = "scatter3d", 
        mode = "markers"
    )
```

Next, we collect performance metrics from the models and compare them. Sidenote: the strength of the tidymodels framework is that it establishes generic, flexible methods for workflows across diverse models. The drawback is that the code to accomplish any given task can get a little complicated (as seen below).

Code

```
rf_results <-
    map2(
        .x = list(rf_all, rf_subset),
        .y = c("rf_all", "rf_subset"),
        .f = 
            ~collect_metrics(.x) |> 
            select(mtry, min_n, .metric, mean) |> 
            pivot_wider(
                id_cols = c(mtry, min_n),
                names_from = .metric,
                values_from = mean,
                names_prefix = "mean_"
            ) |> 
            slice_max(mean_roc_auc, n = 1) |> 
            mutate(model = .y, .before = mtry)
    ) |> 
    reduce(bind_rows)

rf_results |> 
    relocate(mean_roc_auc, .before = mean_accuracy) |> 
    arrange(desc(mean_roc_auc))
```

The random forest model using a subset of predictors performs best on average when evaluated on a set of bootstrapped split samples drawn from the training data. Let’s fit this model on the entire training set of data, then evaluate it on the reserved test set of data (which we have not touched so far):

Code

```
final_model <-
    workflow() |>
    add_formula(subset_pred_spec) |>
    add_model(
        finalize_model(
            rf_spec,
            select_best(rf_subset, metric = "roc_auc")
        )
    ) |>
    last_fit(asthmaster_split)

final_model |> 
    collect_metrics() |> 
    select(.metric, .estimate)
```

The performance results are more or less in line with what we estimated previously with an accuracy of 0.73 of and area under the curve of 0.76.

# Calculate IPW

At long last, we can use this model to calculate inverse probability weights (IPW) for each encounter in the original dataset. Higher IPW values correspond to encounters where the observed dose of continuous albuterol was unexpected based on our model’s predictions. For example, the top row gets a very high IPW because the observed dose was 10 mg/hr, but the model predicted a 93% probability that the dose would be 15 mg/hr based on the year, age, race, initial heart rate, and initial breathing rate of the patient:

Code

```
final_model$.workflow[[1]] |> 
    augment(new_data = asthmaster_compact) |> 
    select(FIN, .pred_15 = `.pred_15 mg/hr`) |> 
    inner_join(asthmaster_modified, by = "FIN") |> 
    mutate(
        ipw = (Albuterol_Cont_Dose_Logit / .pred_15) + ((1 - Albuterol_Cont_Dose_Logit) / (1 - .pred_15)),
        .after = .pred_15
    ) |> 
    select(observed_albuterol_dose = Albuterol_Cont_Dose_Factor, pred_prob_of_15_dose = .pred_15, ipw, Year, AgeAtEncounter, Race_Black, HR_Initial, RR_Initial) |> 
    arrange(desc(ipw))
```
